# ﻿Morphological and molecular evidence reveals three new species of *Lithocarpus* (Fagaceae) from Bidoup-Nui Ba National Park, Vietnam

**DOI:** 10.3897/phytokeys.186.69878

**Published:** 2021-12-06

**Authors:** Nguyen Van Ngoc, Hoang Thi Binh, Ai Nagahama, Shuichiro Tagane, Hironori Toyama, Ayumi Matsuo, Yoshihisa Suyama, Tetsukazu Yahara

**Affiliations:** 1 Faculty of Biology, Dalat University, 01 - Phu Dong Thien Vuong, Dalat, Vietnam Dalat University Dalat Vietnam; 2 Faculty of Science, Kyushu University, 744 Motooka, Fukuoka, 819-0395, Japan Kyushu University Fukuoka Japan; 3 The Kagoshima University Museum, Kagoshima University, 1-21-30 Korimoto, Kagoshima, 890-0065, Japan Kagoshima University Kagoshima Japan; 4 Biodiversity Division, National Institute for Environmental Studies, Tsukuba, Ibaraki, 305-8506, Japan Biodiversity Division, National Institute for Environmental Studies Ibaraki Japan; 5 Kawatabi Field Science Center, Graduate School of Agricultural Science, Tohoku University, 232-3 Yomogida, Naruko-onsen, Osaki, Miyagi 989-6711, Japan Tohoku University Miyagi Japan; 6 Kyushu Open University, 744 Motooka, Fukuoka, 819-0395, Japan Kyushu Open University Fukuoka Japan

**Keywords:** Fagales, Lam Dong Province, MIG-seq, phylogeny, taxonomy

## Abstract

Three new species, *Lithocarpusbidoupensis* Ngoc & Tagane, *L.congtroiensis* Ngoc & Yahara, and *L.hongiaoensis* Ngoc & Binh are described from Bidoup-Nui Ba National Park, Central Highland of Vietnam. Morphological analyses and Maximum likelihood tree based on genome-wide SNPs support the distinction of those species from the previously known taxa in the region. The three new species are considered to be endemic to the Bidoup-Nui Ba National Park and the preliminary conservation status for each species is evaluated as Critically Endangered.

## ﻿Introduction

Fagaceae are highly diversified in Vietnam and 216 species of 6 genera have been reported in various forest types, from dry evergreen forest at lowlands to montane evergreen forest in the higher elevation (Ho 2003; Ban 2005; Ngoc et al. 2016). Recently, 10 species of Fagaceae were newly described from Vietnam: *Castanopsisgrandicicatricata* N.H.Xia & D.H.Vuong, *C.multiporcata* N.H.Xia & D.H.Vuong (Vuong and Xia 2014), *Lithocarpusdahuoaiensis* Ngoc & L.V.Dung (Ngoc et al. 2016), *L.vuquangensis* Ngoc & V.H.Nguyen (Ngoc et al. 2018), *Quercusbaolamensis* H.T.Binh & Ngoc (Binh et al. 2018a), *Q.bidoupensis* H.T.Binh & Ngoc (Binh et al. 2018a), *Q.honbaensis* H.T.Binh, Tagane & Yahara (Binh et al. 2018a), *Q.trungkhanhensis* H.T.Binh & Ngoc (Binh et al. 2018b), *Q.xuanlienensis* H.T.Binh, Ngoc & T.N.Bon (Binh et al. 2018c), and *Q.ngochoaensis* Binh & Son (Binh et al. 2021).

*Lithocarpus* Blume is the largest genus of the family Fagaceae in Vietnam, including 119 species and two varieties, among which 44 species are endemic (Ho 2003; Ban 2005; Linh et al. 2013; Ngoc et al. 2016, 2018). The previous taxonomic treatments of *Lithocarpus* in Vietnam were mostly based on Camus’ studies using the specimens collected by French botanists, the results of which were documented in the part of *Flore générale de l’Indo-Chine* (Hickel and Camus 1930) or *Chênes Atlas* (Camus 1948). All these early studies relied only on morphological features to identify and construct the keys to species level, so the taxonomic circumscriptions were sometimes inaccurate, leading to continued uncertainty in the taxonomic status and relationship among species.

The phylogenetic approach has become a widespread and efficient way to identify and delimit species, but there is only one study for *Lithocarpus* in Vietnam (Ngoc et al. 2018). The MIG-seq is a PCR-based method used to identify large numbers of genetic markers throughout the genome (Suyama and Matsuki 2015) that is highly applicable for use in phylogenetic studies (Binh et al. 2018a; Okabe et al. 2021). Recently, a growing number of taxonomic studies of *Lithocarpus* have yielded new species (Ngoc et al. 2016, 2018), but we often find *Lithocarpus* material that is difficult to identify to species level. Here we applied MIG-seq for phylogenetic reconstruction to accurately assess the diversity and taxonomy of *Lithocarpus* species.

**Figure 1.**
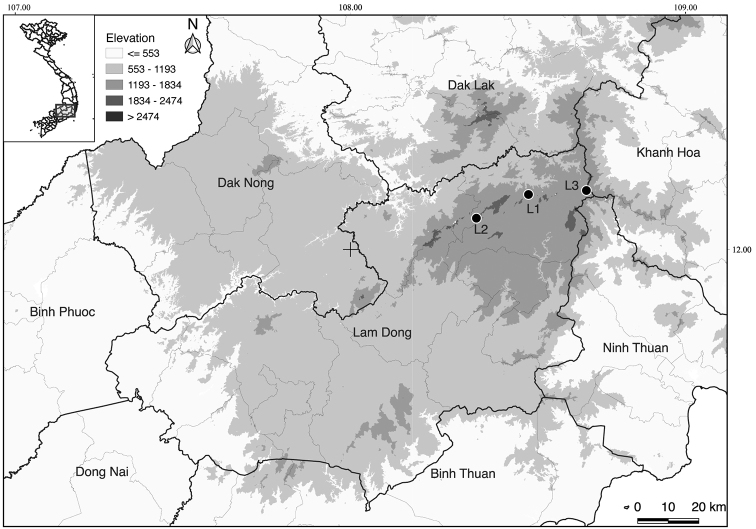
Type locality of the new species (Black dots): L1: *L.bidoupensis*, L2: *L.congtroiensis*, and L3: *L.hongiaoensis*.

Bidoup-Nui Ba National Park (Fig. 1) is in the core zone of UNESCO Langbiang Biosphere Reserve, which is in Lam Dong Province in the central highland of Vietnam. The national park, with the area of 70,038 ha covering almost the entire Langbiang Plateau, harbors 1933 species of vascular plants (Bidoup-Nui Ba National Park 2021) including 62 threatened species (Ban et al. 2007; IUCN 2012), and 42 endemic species (Tagane et al. 2017, 2020; Bidoup-Nui Ba National Park 2021). For Fagaceae, 25 species of *Lithocarpus*, nine species of *Castanopsis*, eleven species of *Quercus*, and one species of *Trigonobalanus* have been recorded from Bidoup-Nui Ba National Park (Dung 2005; Ngoc et al. 2016; Binh et al. 2018a).

During our floristic research in Bidoup-Nui Ba National Park from 2015 to present, we found some individuals of the genus *Lithocarpus* that could not be identified to species level. We here describe them as *Lithocarpusbidoupensis* Ngoc & Tagane, sp. nov., *Lithocarpushongiaoensis* Ngoc & Binh, sp. nov., and *Lithocarpuscongtroiensis* Ngoc & Yahara, sp. nov., based on comparisons of morphology with related species and provide molecular phylogenetic evidence using the MIG-seq method (Suyama and Matsuki 2015).

## ﻿Materials and methods

### ﻿Taxon sampling

In the present study, we conducted botanical inventories in Bidoup-Nui Ba National Park and the other protected areas in Vietnam and collected a total of 63 samples consisting of 23 species including sixteen samples of three unknown species. Five samples of *Lithocarpusbalansae* (Drake) A.Camus, which is morphologically distinct from the other species of the genus, were included as an outgroup for the phylogenetic analysis. Localities and voucher specimens of these materials are listed in Table 1.

**Table 1.** List of vouchers specimen that were used in this study.

Species	Vouchers	Localities
*L.annamitorus*	*Nguyen* et al. *V3214*	Bidoup-Nui Ba NP
*L.bidoupensis*	*Tagane* et al. *V4320, Yahara* et al. *V7850, V8190, V8417, V9940* (DLU, FU, KAG); *Ngoc* et al. *NAF122, NAF185* (DLU)	Bidoup-Nui Ba NP
*L.blaoensis*	*Nguyen* et al. *V3176, V3176A* (DLU, FU)	Bidoup-Nui Ba NP
*L.coalitus*	*Tagane* et al. *V4191, Yahara* et al. *V10140* (DLU, FU),	Bidoup-Nui Ba NP
*L.congtroiensis*	*Ngoc* et al. *NAF200* (DLU); *Nguyen* et al. *V3205, Tagane* et al. *V9102*, *Tagane* et al. *V9470*, *Tagane* et al. *V9492*, *Yahara* et al. *V9555* (DLU, FU)	Bidoup-Nui Ba NP
*L.encleisocarpus*	*Tagane* et al. *V1627; Nguyen* et al. *V3263* (DLU, FU)	Bidoup-Nui Ba NP
*L.dahuoaiensis*	*Nguyen* et al. *V3194* (DLU, FU)	Bidoup-Nui Ba NP
	*Ngoc* et al. *V5404, V5404A* (DLU, FU)	Dong Nai NR
*L.dalatensis*	*Tagane* et al. *V9106* (DLU, FU, KAG)	Bidoup-Nui Ba NP
*L.dealbatus*	*Ngoc* et al. *V3258, Tagane* et al. *V4357* (DLU, FU)	Bidoup-Nui Ba NP
*L.hancei*	*Ngoc* et al. *V5111, V4918, SP008* (DLU, FU)	Hoang Lien NP
*L.honbaensis*	*Tagane* et al. *V0003, V207; Ngoc* et al. *V5540* (DLU, FU)	Hon Ba NR
*L.hongiaoensis*	*Nguyen* et al. *V3235* (DLU, FU); *Ngoc* et al. *NAF123, NAF192* (DLU)	Bidoup-Nui Ba NP
*L.laoticus*	*Nguyen* et al. *V3193* (DLU, FU)	Bidoup-Nui Ba NP
*L.lemeeanus*	*Tagane* et al. *V4523* (DLU, FU)	Bidoup-Nui Ba NP
*L.licentii*	*Tagane* et al. *V6261, V6400* (DLU, FU)	Ngoc Linh NR
*L.* sp1	*Nguyen* et al. *V3171, V3171A* (DLU, FU)	Bidoup-Nui Ba NP
*L.parvulus*	*Yahara* et al. *V8636, V9720, V10068, V10077, V10164* (DLU, FU, KAG)	Bidoup-Nui Ba NP
*L.pseudomagneinii*	*Nguyen* et al. *V3183, V3223* (DLU, FU)	Bidoup-Nui Ba NP
*L.syncarpus*	*Nguyen* et al. *V3188, V3188A, V3246, V3250* (DLU, FU)	Bidoup-Nui Ba NP
*L.vinhensis*	*Nguyen* et al. *V3591, V3787* (DLU, FU)	Vu Quang NP
*L.vuquangensis*	*Yahara* et al. *V5743, V5938* (DLU, FU)	Vu Quang NP
*L.xylocarpus*	*Tagane* et al. *V4337; Ngoc* et al. *V8464* (DLU, FU)	Bidoup-Nui Ba NP
*L.balansae* (outgroup)	*Yahara* et al. *V2938* (DLU, FU)	Bach Ma NP
	*Nguyen* et al. *V3177, Ngoc* et al. *V8467* (DLU, FU)	Bidoup NP
	*Nguyen* et al. *V5447, V5512* (DLU, FU)	Pu Mat NP

NP = National Park; NR = Nature Reserve.

### ﻿Morphological analysis

We compared morphological traits of three unknown species with those of related species using taxonomic literature (Camus 1931, 1938, 1942, 1943, 1945, 1948; Huang et al. 1999; Ho 2003; Ban 2005; Phengklai 2008), specimens kept in the herbaria ANDA, BKF, DLU, HN, KAG, KYO, P, and VNM, and digitized plant specimen images available on the web of JSTOR Global Plants (https://plants.jstor.org/) and Chinese Virtual Herbarium (http://www.cvh.org.cn/).

The ImageJ software (Schneider et al. 2012) was used to measure the following characters of the new species and related species based on images of type specimens: length, width, aspect ratio and circularity of leaf blade, petiole length, and size of cupules. Aspect ratio and circularity are defined as length/width of leaf blade and 4π × (area/perimeter squared), respectively. Analysis of variance (ANOVA) and post hoc Tukey’s honestly significant difference test (Tukey’s HSD) (Tukey 1953) were applied to reveal the mean difference among species. All statistical analyses were performed in R version 4.0.5 (R Core Team 2021) with R-Sutido ver. 1.4.1106 (R-Studio Team 2021).

### ﻿DNA extraction and sequencing

Leaf pieces were dried using silica-gel in the field, and DNA was isolated with the CTAB method (Doyle and Doyle 1987) with minor modifications described in Toyama et al. (2015). The extracted DNA was diluted to 10 ng/µl and used as templates to amplify thousands of short sequences (loci) from a wide variety of genomes with a standard PCR protocol according to Suyama and Matsuki (2015). MIG-seq library was constructed as described in Suyama and Matsuki (2015) with a minor update by using dual-indexed primers (Suyama et al. 2021). The 1^st^ PCR, multiple non-repetitive regions from various inter-simple-sequence repeats (ISSRs) were amplified from genomic DNA by multiplexed PCR with tailed ISSR forward and reverse primers sets. The first PCR products were diluted and used as the templates for the 2^nd^ PCR with dual indexed primers sets. Then, 3 µl of each 2^nd^ PCR product was pooled in equimolar concentrations as single mixture library. The mixture was then purified and the size range of 350–800 bp were isolated by a Pippin Prep DNA size selection system (Sage Science, Beverly, MA, USA). Quantitative PCR was performed to measure final concentration of size-selected library with approximately 10 pM and then used for sequencing on an Illumina MiSeq Sequencer (Illumina, San Diego, CA, USA), using a MiSeq Reagent Kit v3 (150 cycle, Illumina).

### ﻿Phylogenetic analysis

A total of 50 samples of 22 species of *Lithocarpus* including samples of unknown species were sequenced (except *NAF122*, *NAF123*, *NAF185*, *NAF192*, *V3205*, *V9470*, *V9492*, *V9555*), of which five samples of *L.balansae* were used as an outgroup. The low-quality reads and primer sequences were eliminated from raw data by using the trimmomatic software version 0.40 (Bolger et al. 2014). The quality-filtered sequence data were demultiplexed and filtered through the software Stacks v1.46 (Catchen et al. 2011; Catchen et al. 2013) following the parameters set as described by Takata et al. (2019) with minor modifications: in the U-stacks, the option settings of ‘maximum distance allowed between stacks (M)’ = 4,‘maximum distance allowed to align secondary reads to primary stacks (N)’ = 4; in the population program, the minimum percentage of individuals required to process a locus across all data (r) was set at 10% and the minimum minor allele frequency required to process a nucleotide site at a locus (min_maf) = 0.005, the maximum observed heterozygosity required to process a nucleotide site at a locus (max_obs_het) = 0.6.

Phylogenetic analyses were conducted using maximum likelihood method on SNPs data set. The model of sequence evolution was set to GTR+G as selected by jMrModeltest 2.1.10 (Darriba et al. 2012). Maximum Likelihood analyses were implemented using the RAxML ver. 8.2 (Stamatakis 2014). The topological reliability of the maximum likelihood tree was evaluated with 1000 bootstrap replicates.

## ﻿Results

### ﻿Morphological analyses

After the morphological examination and taxonomic review in *Lithocarpus* of Vietnam and its surrounding countries, sixteen unknown samples of *Lithocarpus* were not assignable to any of the species recognized in the region. Hence, hereafter we named these samples as (1) *Lithocarpusbidoupensis* Ngoc & Tagane, sp. nov. for *Ngoc* et al. *NAF125*, *NAF185*; *Tagane* et al. *V4320*; *Yahara* et al. *V7850*, *V8190*, *V8417*, and *V9940*; (2) *Lithocarpuscongtroiensis* Ngoc & Yahara, sp. nov. for *Ngoc* et al. *NAF200*; *Nguyen* et al. *V3205*, *Tagane* et al. *V9102*, *V9470*, *V9492*; *Yahara* et al. *V9555*; and (3) *Lithocarpushongiaoensis* Ngoc & Binh, sp. nov. for *Nguyen* et al. *V3235*; *Ngoc* et al. *NAF123*, *NAF192*.

*Lithocarpusbidoupensis* is most similar to *L.blaoensis* in having completely entire leaf margin, leaf blade width 3–5 cm, 10–12 pairs of secondary veins, cupules clustered in sets of three, and a concave nut scar, but ANOVA with a post-hoc Tukey HSD test showed significant differences (p < 0.05) between species (Table 2 and Table 3). Specifically, *L.bidoupensis* significantly differed in much shorter petioles (0.5 ± 0.1 cm long in *L.bidoupensis* vs. 1.89 ± 0.23 cm long in *L.blaoensis*), shorter leaf blades (9.74 ± 1.12 cm long vs. 13.66 ± 1.89 cm long), bigger cupules (0.98 ± 0.19 cm high, 2.47 ± 0.2 cm in diam. vs. 0.64 ± 0.06 cm high, 1.58 ± 0.11 cm in diam.) *Lithocarpusbidoupensis* also has a larger scar of the nut than *L.blaoensis* (1.4–1.9 cm in diam. vs. 1–1.2 cm in diam.). *Lithocarpusbidoupensis* is also similar to *L.licentii*. Both have a completely entire leaf margin, glossy green leaf blades, a leaf blade length of 7.2–11.6 cm, and a cupule height of 0.7–1.4 cm. However, *L.bidoupensis* has a significantly shorter and wider leaf blade (9.74 ± 1.12 cm × 4.5 ± 0.59 cm in *L.bidoupensis* vs. 10.76 ± 2.01 cm × 3.46 ± 0.53 cm in *L.licentii*), lower leaf blade aspect ratio (2.17 ± 0.15 vs. 3.1 ± 0.31), higher leaf blade circularity (0.71 ± 0.03 vs. 0.55 ± 0.05), shorter petioles (0.5 ± 0.1 vs. 0.81 ± 0.14), and bigger cupules size (0.98 ± 0.19 cm cm high, 2.47 ± 0.2 cm in diam. vs. 0.88 ± 0.15 cm cm high, 2.17 ± 0.13 cm in diam.) (Table 2 and Table 3). It also has fewer secondary veins (10–12 pairs in *L.bidoupensis* vs. 12–15 pairs in *L.licentii*), much shorter infructescences (8.4–11.5 cm long vs. 15–20 cm long), cupules clustered in sets of three (vs. solitary in *L.licentii*), a cupule covering less than 1/3 of the nut (vs. covering 1/2–2/3 of the nut), and a concave basal scar (vs. convex).

**Table 2.** The comparisons of mean (X) and standard deviation (SD) value of the leaf blade and cupule size between *L.bidoupensis*, *L.congtroiensis*, and *L.hongiaoensis* with related species.^1^Derived from type specimens, ^2^Derived from this study collections, n = number of leaf or cupule were measured in this study.

Parameters (cm)	*L.bidoupensis ^1^*			*L.blaoensis ^1,2^*			*L.licentii ^1,2^*		
	X	SD	n	X	SD	n	X	SD	n
Leaf blade length	9.74	1.12	23	13.66	1.89	22	10.76	2.01	20
Leaf blade width	4.5	0.59	23	4.41	0.51	22	3.46	0.53	20
Leaf blade aspect ratio	2.17	0.15	23	3.11	0.36	22	3.1	0.31	20
Leaf blade circularity	0.71	0.03	23	0.58	0.05	22	0.55	0.05	20
Petiole length	0.5	0.1	23	1.89	0.23	22	0.81	0.14	20
Cupule high	0.98	0.19	27	0.64	0.06	22	0.88	0.15	16
Cupule diameter	2.47	0.2	27	1.58	0.11	22	2.17	0.13	16
	***L.congtroiensis ^1^***			***L.dahuoaiensis ^1,2^***			***L.honbaensis ^1,2^***		
	**X**	**SD**	**n**	**X**	**SD**	**n**	**X**	**SD**	**n**
Leaf blade length	14.83	1.6	22	19.4	3.45	22	20.39	3.44	20
Leaf blade width	5.3	0.84	22	8.06	1.48	22	8.84	1.66	20
Leaf blade aspect ratio	2.86	0.34	22	2.41	0.17	22	2.32	0.16	20
Leaf blade circularity	0.59	0.06	22	0.7	0.04	22	0.69	0.04	20
Petiole length	1.42	0.19	22	1.4	0.14	22	2.11	0.36	20
Cupule high	1.10	0.18	21	1.23	0.14	28	-	-	-
Cupule diameter	2.99	0.28	21	2.24	0.19	28	-	-	-
	***L.hongiaoensis ^1^***			***L.vinhensis ^1,2^***			***L.vuquangensis ^1,2^***		
	**X**	**SD**	**n**	**X**	**SD**	**n**	**X**	**SD**	**n**
Leaf blade length	10.81	1.93	29	8.42	2.26	22	7.49	1.32	25
Leaf blade width	3.26	0.6	29	2.97	0.95	22	2.39	0.32	25
Leaf blade aspect ratio	3.33	0.33	29	2.9	0.4	22	3.13	0.33	25
Leaf blade circularity	0.49	0.04	29	0.57	0.07	22	0.5	0.05	25
Petiole length	2.59	0.49	29	0.74	0.12	22	1.3	0.23	25
Cupule high	1.01	0.15	25	0.57	0.05	19	0.86	0.26	18
Cupule diameter	2.06	0.28	25	1.31	0.14	19	1.63	0.18	18

*Lithocarpuscongtroiensis* is morphologically similar to *L.dahuoaiensis* in having a completely entire leaf margin, blade broadly elliptic, glabrous adaxially, petioles length 1–1.8 cm long, and concave nut scar, but significantly differed in shorter leaf blades (14.83 ± 1.6 cm × 5.3 ± 0.84 cm in *L.congtroiensis* vs. 19.4 ± 3.45 cm × 8.06 ± 1.48 cm in *L.dahuoaiensis*), higher leaf blades aspect ratio (2.86 ± 0.34 vs. 2.41 ± 0.17), lower leaf blade circularity (0.59 ± 0.06 vs. 0.7 ± 0.04), bigger cupules (2.99 ± 0.28 vs. 2.24 ± 0.19) (Table 2 and Table 3), and also differs in having more secondary veins (13–15 pairs vs. 11–12 pairs), shorter infructescences (10–15 cm long vs. 20–25 cm long), cupule clustered of three (vs. solitary). *Lithocarpuscongtroiensis* is also similar to *L.honbaensis* in having completely entire leaf margin, adaxially glabrous lamina, long petioles, cupule clustered of three, and concave nut scar, but significantly differed in smaller leaf blades (14.83 ± 1.6 cm × 5.3 ± 0.84 cm in *L.congtroiensis* vs. 20.39 ± 3.44 cm × 8.84 ± 1.66 cm in *L.honbaensis*), higher leaf blade aspect ratio (2.86 ± 0.34 vs. 2.32 ± 0.16), lower leaf blade circularity (0.59 ± 0.06 vs. 0.69 ± 0.04), shorter petioles (1.42 ± 0.19 vs. 2.11 ± 0.36) (Table 2 and Table 3), and also differed in having more secondary veins (13–15 pairs vs. 10–11 pairs), shorter infrutescences (10–15 cm long vs. 15–24 cm long), and shorter acorn-stalk (0.2–0.4 cm long vs. 0.5–0.8 cm long).

**Table 3.** Differences between the species for morphological characters and their levels of significance determined by TukeyHSD Test.

Comparisons	Leaf blade length		Leaf blade width		Aspect ratio		Circularity		Petiole length		Cupule high		Cupule diameter	
	diff.	*P*	diff.	*P*	diff.	*P*	diff.	*P*	diff.	*P*	diff.	*P*	diff.	*P*
*L.bidoupensis* – *L.blaoensis*	3.92	**0**	-0.09	0.85	0.93	**0**	-0.13	**0**	1.39	**0**	-0.35	**0**	-0.89	**0**
*L.bidoupensis* – *L.licentii*	1.01	0.13	-1.04	**<0.001**	0.90	**0**	-0.16	**0**	0.31	**0**	-0.1	0.07	-0.29	**0**
*L.congtroiensis* – *L.dahuoaiensis*	4.57	**<0.001**	2.76	**0**	-0.45	**<0.001**	0.11	**0**	-0.02	0.97	0.13	**<0.01**	-0.75	**0**
*L.congtroiensis* – *L.honbaensis*	5.56	**<0.001**	3.53	**0**	-0.09	**0**	0.09	**0**	0.69	**0**	-	-	-	-
*L.hongiaoensis* – *L.vinhensis*	-2.39	**<0.001**	-0.29	0.26	-0.43	**<0.001**	0.08	**<0.001**	-1.85	**0**	-0.44	**0**	-0.75	**0**
*L.hongiaoensis* – *L.vuquangensis*	-3.33	0	-0.58	**<0.001**	-0.20	0.09	0.02	0.55	-1.29	**0**	-0.15	**0.012**	-0.43	**<0.001**

Note: diff. = mean difference; **Bold** font indicates statistically significant differences, (-) not available.

*Lithocarpushongiaoensis* is most similar to *L.vinhensis* in having blades narrowly elliptic to lanceolate, completely entire leaf margins, adaxially glabrous and abaxially hairy leaf surface, solitary cupules, and concave nut scar, but ANOVA analysis with post-hoc Tukey HSD test showed that *L.hongiaoensis* significantly differed from *L.vinhensis* in having longer leaf blade length (10.81 ± 1.93 cm long in *L.hongiaoensis* vs. 8.42 ± 2.26 cm long in *L.vinhensis*), higher leaf blade aspect ratio (3.33 ± 0.33 vs. 2.9 ± 0.4), lower leaf blade circularity (0.49 ± 0.04 vs. 0.57 ± 0.07), much longer petioles (2.59 ± 0.49 vs. 0.74 ± 0.12), bigger cupules (1.01 ± 0.15 cm high, 2.06 ± 0.28 cm in diam. vs. 0.57 ± 0.05 cm high, 1.31 ± 0.14 cm in diam.) (Table 2 and Table 3). *Lithocarpushongiaoensis* is morphologically almost identical with *L.vuquangensis* such as blade narrowly elliptic to lanceolate, completely entire leaf margin, midrib flat or slightly prominent, adaxially glabrous and abaxially hairy leaf surface, solitary cupules, and concave nut scar, but the new species significantly differed in having bigger leaf blades (10.81 ± 1.93 cm × 3.26 ± 0.6 cm in *L.hongiaoensis* vs. 7.49 ± 1.32 cm × 2.39 ± 0.32 cm in *L.vuquangensis*), longer petioles (2.59 ± 0.49 vs. 1.3 ± 0.23), bigger cupules (1.01 ± 0.15 cm high, 2.06 ± 0.28 cm in diam. vs. 0.86 ± 0.26 cm high, 1.63 ± 0.18 cm in diam.), and also differs in having longer infrutescences (12.5–16.5 cm long vs. 5–7 cm long), larger nut scar (1.2–1.4 cm in diam. vs. 1–1.1 cm in diam.) (Table 2 and Table 3).

### ﻿Phylogeny inference

**Figure 2.**
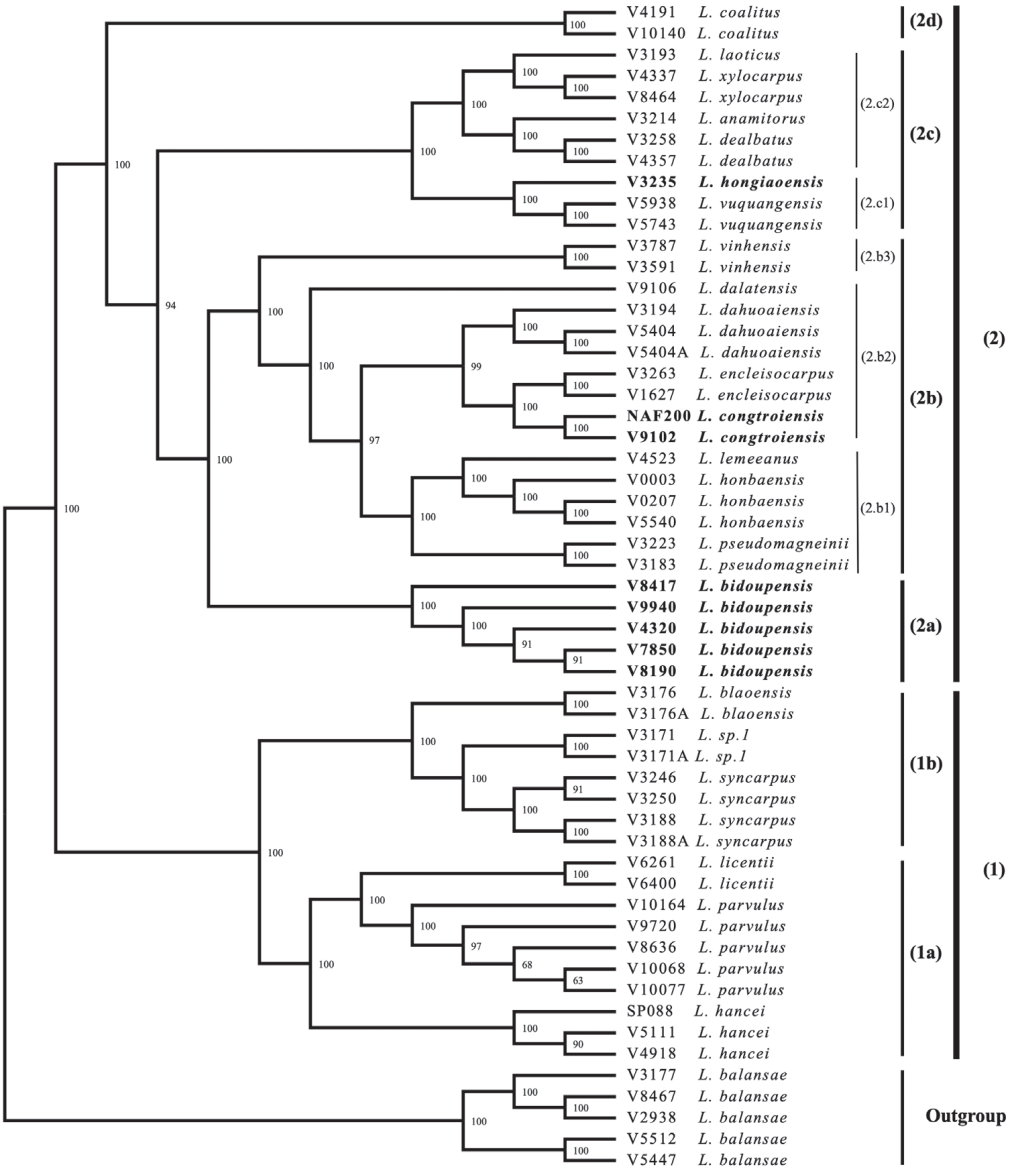
Maximum likehood tree of three new species (**Bold**) with their related based on SNPs data from MIG-seq.

The Maximum likelihood tree based on data set of 4962 genome-wide SNPs strongly supports two sister clades, clade 1 and 2, with 100% bootstrap value (Fig. 2). Clade 1 is divided into two subclades, 1a and 1b, each with 100% bootstrap value. Clade 2 is divided into four subclades of 2a, 2b, 2c, and 2d, each with 100% bootstrap value. The three candidates for new species were included in the clade 2 and supported the monophyly of *L.congtroiensis* and *L.bidoupensis* with 100% bootstrap value. The specimen here described as *Lithocapushongiaoensis* is sister to two specimens of *L.vuquangensis*.

*Lithocarpusbidoupensis* (clade 2a) was clearly separated from the morphologically similar species of *L.licentii* (Clade 1a) and *L.blaoensis* (Clade 1b) and is sister to many different species in clade 2b. *Lithocarpuscongtroiensis*is is included in a clade with *L.encleisocarpus*, *L.lemeeanus* and *L.pseudomagneinii* (clade 2.b2) with 97% bootstrap support, but it is well supported as monophyletic The morphologically similar *L.honbaensis* is in a different clade (clade 2.b1).

*Lithocarpushongiaoensis* (clade 2.c1) was clearly separated from the morphologically similar species of *L.vinhensis* (clade 2.b3) but showed a sister relationship with *L.vuquangensis* in the clade 2.c1 with a strongly bootstrap value (100%). Those three species share the character of solitary cupules but the distribution of *L.hongiaoensis* is narrowly restricted and apart from the two species: *L.vinhensis* and *L.vuquangensis* are distributed in Nghe An and Ha Tinh Provinces, which are located in the north of the Central Coast of Viet Nam, whereas *L.hongiaoensis* was found only in Bidoup-Nui Ba National Park, Lam Dong Province about 1000 km further south.

## ﻿Discussion

The morphological comparison and phylogenetic analysis provided evidence of the validity of three new species. *Lithocarpusbidoupensis* is most similar to *L.blaoensis* that occurred in the same locality with *L.bidoupensis*, and also similar to *L.licentii* that was collected in Kon Tum Province, which is the type locality of *L.licentii*. However, the new species is clearly different from both in many morphological traits (Table 2 and Table 3) as well as shown in the phylogenetic results (Fig. 2). The molecular phylogenetic tree strongly supports this disjunction in that the monophyly of *L.bidoupensis* was supported by 100% bootstrap value, while *L.blaoensis* and *L.licentii* were placed in another clade.

*Lithocarpuscongtroiensis* is placed in the same clade with *L.encleisocarpus* and *L.dahuoaiensis* (2.b2), but the morphology was clearly distinct. *Lithocarpuscongtroiensis* is distinguished from *L.encleisocarpus* by its greater number of secondary vein (13–15 pairs in *L.congtroiensis* vs. 8–10 pairs in *L.encleisocarpus*), cupules with tiny imbricate scales, enclosing 1/3–1/2 of the nut (vs. the scales forming 5–7 dimly concentric flanges, cupules completely enclosing the nut). Especially, the cupule of *L.congtroiensis* usually clustered of three, while the cupules of *L.encleisocarpus* is solitary. The morphological distinctness between *L.congtroiensis* and *L.dahuoaiensis* is clearly shown in Tables 2 and 3.

Although we could not collect any specimens of *L.honbaensis* with mature fruits, the morphological analysis of leaf and cupule characters provided enough evidence to distinguish species from *L.congtroiensis*. In addition, the molecular phylogenetic tree showed that *L.honbaensis* has a close genetic relationship with *L.lemeeanus* and *L.pseudomagneinii* than *L.congtroiensis* (clade 2b).

*Lithocarpushongiaoensis* is most similar to *L.vinhensis* and *L.vuquangensis*, of which the latter showed the sister relationship to *L.hongiaoensis* in the molecular phylogenetic tree (Fig. 2, clade 2.c1). However, *L.hongiaoensis* is narrowly endemic to the Hon Giao area of Bidoup-Nui Ba National Park, Lam Dong Province, in the southern part of Vietnam while *L.vuquangensis* is endemic to Vu Quang National Park of Ha Tinh Province, north-central coast of Vietnam. From 2015 to 2017 we conducted three field trips at Bach Ma National Park, Ba Na Nature Reserve and Ngoc Linh National Park. These protected areas are located between Lam Dong and Ha Tinh Province, but we did not find any individual of *Lithocarpus* similar to *L.hongiaoensis* or *L.vuquangensis*. Also, morphological differences are distinct enough to distinguish them as different species (Table 2 and 3). While *L.hongiaoensis* was collected in Lam Dong Province, *L.vinhensis* occurred in Nghe An Province, the province located in the north central coast of Vietnam. The genetic differences between *L.hongiaoensis* and *L.vinhensis* were presented in the phylogenetic tree, *L.hongiaoensis* and *L.vuquangensis* a sister to each other (2.c1), while the two samples of *L.vinhensis* formed a clade not closely related to these two species (2.b3).

### ﻿Taxonomic treatments

#### ﻿ Lithocarpus bidoupensis

Taxon classificationPlantaeFagalesFagaceae

Ngoc & Tagane sp. nov.

DB34B140-DE33-5C86-A085-8132D26A1EB9

urn:lsid:ipni.org:names:77234073-1

Fig. 3

##### Type.

Vietnam. Lam Dong Province: Bidoup-Nui Ba National Park, in hill evergreen forest dominated by the species of Fagaceae, 1698 m elev., 12°09'52.95"N, 108°32'00.38"E, 24 February 2016, *S. Tagane*, *H. Toyama*, *H. Nagamasu*, *A. Naiki*, *V.S. Dang*, *N.V. Ngoc*, *J. Wai V4320* [fr.] (holotype DLU!, isotypes FU!, HN!, KYO!, VNM!).

##### Diagnosis.

*Lithocarpusbidoupensis* is most similar to *L.blaoensis* but differs in its shorter leaf blades, petioles and infructescences, and bigger cupules and nuts. The new species is also similar to *L.licentii* but distinguished from *L.licentii* by its shorter petioles, fewer secondary veins (10–12 pairs in *L.bidoupensis* vs. 12–15 pairs in *L.licentii*), much shorter infructescences, clustered cupules (vs. solitary), cupule covering less than 1/3 of the nut (vs. 1/2–2/3 of the nut), and concave basal scar of the nut (vs. convex) (Table 2).

**Figure 3.**
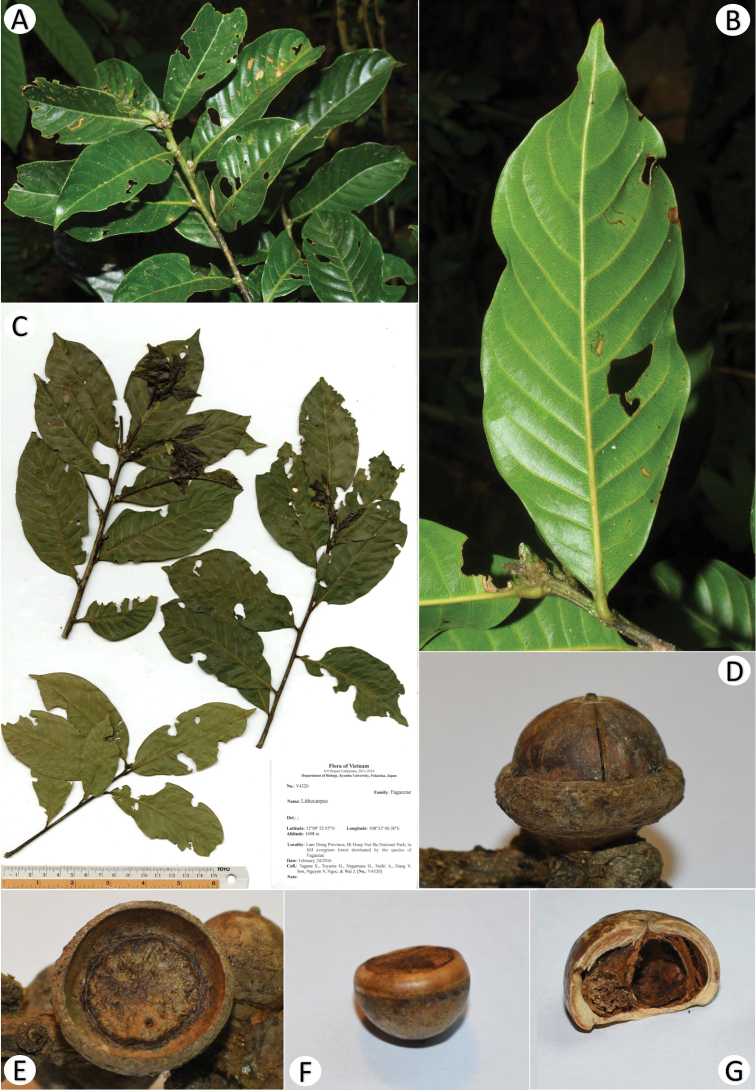
*Lithocarpusbidoupensis* Ngoc & Tagane **A** leafy twig **B** abaxial leaf surface **C** holotype (*Tagane* et al. *V4320*, DLU) **D** mature fruit **E** cupule **F** mature nut **G** section of mature nut.

##### Description.

Evergreen tree, up to 27 m tall. Branches yellowish green when young, turning greyish brown when old, glabrous, sparsely lenticellate. Terminal and lateral buds ovoid, up to 7 mm long. Leaves alternate; petiole 0.3–0.4 cm long, glabrous; blade elliptic to oblong-elliptic, obovate-elliptic, 6–11.6 × 2.8–5.3 cm, coriaceous, glabrous, glossy green on both surfaces, apex acuminate or attenuate, acumen up to 0.9 cm long, base cuneate, rarely obtuse, margin completely entire, midrib prominent on both surfaces, secondary veins 10–12 pairs, prominent abaxially, at an angle of 55–70° from the midrib, tertiary veins scalariform-reticulate, visible abaxially. Inflorescences not seen. Infructescences a woody spike, 8.4–11.5 cm long. Cupules sessile, usually in cluster of 3, fused at the base each other, depressed obconical or saucer-shaped, 0.71–1.40 cm high, 2.13–2.80 cm in diam., enclosing 1/4–1/3 of the nut, pubescent with short grayish indumentum outside; wall woody, ca. 2 mm thick, with brown triangular scales outside, the scales up to 4 × 4 mm, imbricate, arranged in 3 or 4 interrupted concentric rings. Nut broadly ovoid-conical to depressed ovoid-globose, 1.5–1.6 cm high, 2.1–2.3 cm in diam., glabrous, brown to blackish brown; basal scar slightly concave, 1.4–1.9 cm in diam.

##### Phenology.

Unknown. Fallen fruits were collected in February.

##### Distribution.

Vietnam (Khanh Hoa and Lam Dong provinces) (Fig. 1).

##### Etymology.

The specific epithet is derived from the type locality, Bidoup-Nui Ba National Park, Lam Dong Province, Vietnam.

##### Vernacular name.

Dé đá Bidoup

##### Additional specimens examined.

Vietnam. Lam Dong Province: Bidoup-Nui Ba National Park; 1602 m elev., 12°09'27.6"N, 108°32'06.6"E, 24 Mar. 2018, *T. Yahara*, *H. Nagamasu*, *H. Toyama*, *M. Zhang*, *A. Nagahama*, *N.V. Ngoc*, *K. Tsuchiya V7850* [ster.] (DLU!, FU!); ibid., 1656 m elev., 12°09'36.61"N, 108°32'11.16"E, 24 Mar. 2018, *T. Yahara, S. Tagane, M. Zhang, A. Nagahama, K. Tsuchiya, N.V. Ngoc, H.T. Binh, T.Q. Cuong V8190*, *V8417* [ster.] (DLU!, FU!); ibid., 1669 m elev., 12°09'36.65"N, 108°32'11.18"E, 29 Apr. 2019, *N.V. Ngoc*, *H.T. Binh*, *N.V. Duy*, *T.T. Nhung NAF122* [ster.] (DLU!); ibid., 1669 m elev., 12°09'36.62"N, 108°32'11.25"E, 29 Apr. 2019, *N.V. Ngoc, H.T. Binh, N.V. Duy, T.T. Nhung NAF185* [ster.] (DLU!). Khanh Hoa Province: Son Thai Commune, Khanh Vinh District, in evergreen; 1430 m elev., 12°10'42.09"N, 108°43'32.59"E, 23 Apr. 2019, *T. Yahara*, *S. Tagane*, *A. Nagahama*, *N. Komada*, *V.N. Ngoc*, *H.V. Thanh V9940* [ster.] (DLU!, FU!).

##### Conservation status.

Critically Endangered (CR). From our intensive field survey in Bidoup-Nui Ba Naitonal Park and its vicinities from 2015 to present (Tagane et al. 2017 & 2020, Binh et al 2018b), *Lithocarpusbidoupensis* was found only in a narrow area within the protected areas of Bidoup-Nui Ba National Park, and its adjascent area of Son Thai Commune at the elevation range between 1400 and 1669 m. In the area, we observed fewer than 50 mature individuals. Based on criterion D of the IUCN Red List criteria (IUCN 2012), this species is qualified as CR.

#### ﻿ Lithocarpus congtroiensis

Taxon classificationPlantaeFagalesFagaceae

Ngoc & Yahara sp. nov.

4363A028-4C48-505B-9DAF-812FD36B5FC4

urn:lsid:ipni.org:names:77234074-1

Fig. 4

##### Type.

Vietnam. Lam Dong Province, Bidoup-Nui Ba National Park: Cong Troi, at edge of evergreen forest, roadside, 1752 m elev., 12°05'37.3"N, 108°22'38.8"E, 11 July 2018, *S. Tagane*, *A. Nagahama*, *K. Tsuchiya*, *N.V. Ngoc.*, *T.Q. Cuong V9102* [fr.] (holotype DLU!; isotypes FU!, HN!, KYO!, VNM!).

##### Diagnosis.

*Lithocarpuscongtroiensis* is most similar to *L.dahuoaiensis* but differs by its smaller leaf blades, more secondary veins, shorter infructescences, cupules clustered of three (vs. solitary), and bigger nut size. It is also similar to *L.honbaensis* but distinguished mainly by its shorter petioles, smaller leaf blade, more secondary veins, shorter infructescences, and shorter fruiting stalk (Table 2).

**Figure 4.**
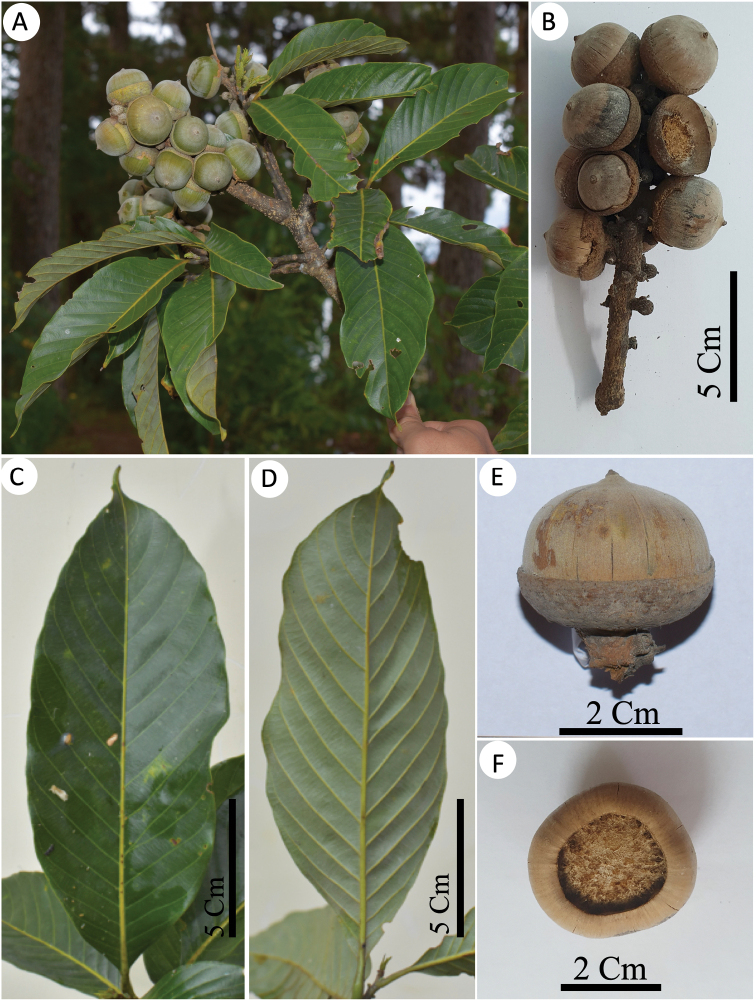
*Lithocarpuscongtroiensis* Ngoc & Yahara **A** twig with infructescence **B** infructescence **C, D** leaves adaxially and abaxially, respectively **E** side view of mature cupule and nut **F** botton view of nut with basal scar.

##### Description.

Evergreen tree, up to 25 m tall. Twigs blackish gray, glabrescent. Stipules caducous, not seen. Leaves alternate; petioles 1.1–1.8 cm long, glabrous; blades broadly elliptic, 12–18 × 4.2–7.2 cm, coriaceous, glabrous on both sides, apex acuminate, acumen 0.7–1.2 cm long, base acute to cuneate, margin entire, midrib flat or slightly prominent adaxially, strongly prominent abaxially, yellowish *in vivo*, brown *in sicco*, secondary veins 13–15 pairs, prominent abaxially, at an angle of 40–50° from the midrib, tertiary veins parallel, faintly visible abaxially. Inflorescence a terminal or axillary spike, 8–12 cm long, erect, male and female flowers separate or female below. Male flowers white, in 1–3-flowered cluster, calyx 6-lobed, lobes ovate, 0.4–0.7 mm × 0.5–0.7 mm; stamens 12, 1–1.2 mm long, anthers 0.15–0.20 mm long. Female flower always cluster of three, styles 3, stigmata pointed. Infructescences terminal, erect, 10–15 cm long, rachis grayish brown, lenticellate. Cupules clustered of three, 0.2–0.4 cm long stalked, bowl-shaped, 0.7–1.4 cm high, 2.5–3.6 m diam., enclosing 1/3–1/2 of the nut; wall woody, with tiny imbricate scales; scales triangular, obscure, covered with white-grayish indumentum. Nut broadly conical or globose, 2.1–2.6 cm high, 2.3–3.1 cm in diam., outer surface densely white tomentose; wall woody, crackled; apex shortly acuminate; basal scar slightly concave, 1.5–1.8 cm in diam.

##### Phenology.

Flowers were collected in December and mature fruits were collected from June to July.

##### Distribution.

Vietnam (so far known only from Mt. Cong Troi and Mt. Langbiang of Bidoup-Nui Ba National Park, Lam Dong Province). (Fig. 1)

##### Etymology.

The specific epithet is derived from the type locality, Mt. Cong Troi of Bidoup-Nui Ba National Park, Lam Dong Province, Vietnam.

##### Vernacular name.

Dé đá Công TrÓi.

##### Additional specimens examined.

Vietnam. Lam Dong Province: Bidoup-Nui Ba National Park, Cong Troi area; 1750 m elev., 12°04'08.5"N, 108°21'55.5"E, 18 June 2015, *N. Nguyen*, *D. Luong*, *B. Hoang V3205* [fr.] (DLU!, FU!); ibid., 1860 m elev., 12°06'06.85"N 108°23'00.32"E, *S. Tagane*, *T. Yahara*, *A. Nagahama*, *M. Zhang*, *K. Tsuchiya*, *T. Nguyen*, *C.T. Nguyen V9470* [fr.] (DLU!, FU!); 1790 m elev., 12°06'03.86"N, 108°23'39.73"E, 20 Dec. 2018, *T. Yahara*, *S. Tagane*, *A. Nagahama*, *K. Tsuchiya*, *C.T. Quong*, *P. Chhang V9555* [male and female fl.] (DLU!, FU! KAG [KAG127308]!); ibid., 1864 m elev., 12°04'08"N, 108°21'54.5"E, 15 Jun. 2019, *N.V. Ngoc, H.T. Binh, N.V. Duy, T.T. Nhung NAF200* [fr.] (DLU!); Mt. Langbiang; 1918 m elev., 7 Oct. 2018, 12°02'48.13"N, 108°26'06.67"E, *S. Tagane*, *T. Yahara*, *A. Nagahama*, *M. Zhang*, *K. Tsuchiya*, *T. Nguyen*, *C.T. Nguyen V9492* [ster.] (DLU!, FU!).

##### Conservation status.

Critically Endangered (CR). We found around ten individuals of *L.congtroiensis* along the road and inside the permanent plot at Cong Troi area, and three individuals in Mt. Langbiang, both located inside the protected area of Bidoup-Nui Ba National Park. Based on criterion D of the IUCN Red List criteria (IUCN 2012), this species is qualified as CR. The new species is endemic to Bidoup-Nui Ba National Park, Lam Dong Province.

#### ﻿ Lithocarpus hongiaoensis

Taxon classificationPlantaeFagalesFagaceae

Ngoc & Binh sp. nov.

98709353-74AD-516F-86E6-E8690B6B8069

urn:lsid:ipni.org:names:77234075-1

Fig. 5

##### Type.

Vietnam. Lam Dong Province, Bidoup-Nui Ba National Park, Hon Giao, at edge of evergreen forest, roadside, 1580 m elev., 12°10'35.9"N, 108°42'25.1"E, 19 June 2015, *N. Nguyen*, *D. Luong*, *B. Hoang V3235* [young male fl. and fruits] (holotype DLU!; isotypes FU!, HN!, KYO!, VNM!).

##### Diagnosis.

*Lithocarpushongiaoensis* is similar to *L.vinhensis* but differs in having much longer petioles, fewer secondary veins, longer infructescences, bigger cupules, and bigger nuts. It is also similar to *L.vuquangensis* but differs in having much longer petioles, more secondary veins, longer infructescences, and bigger cupules (Table 2).

**Figure 5.**
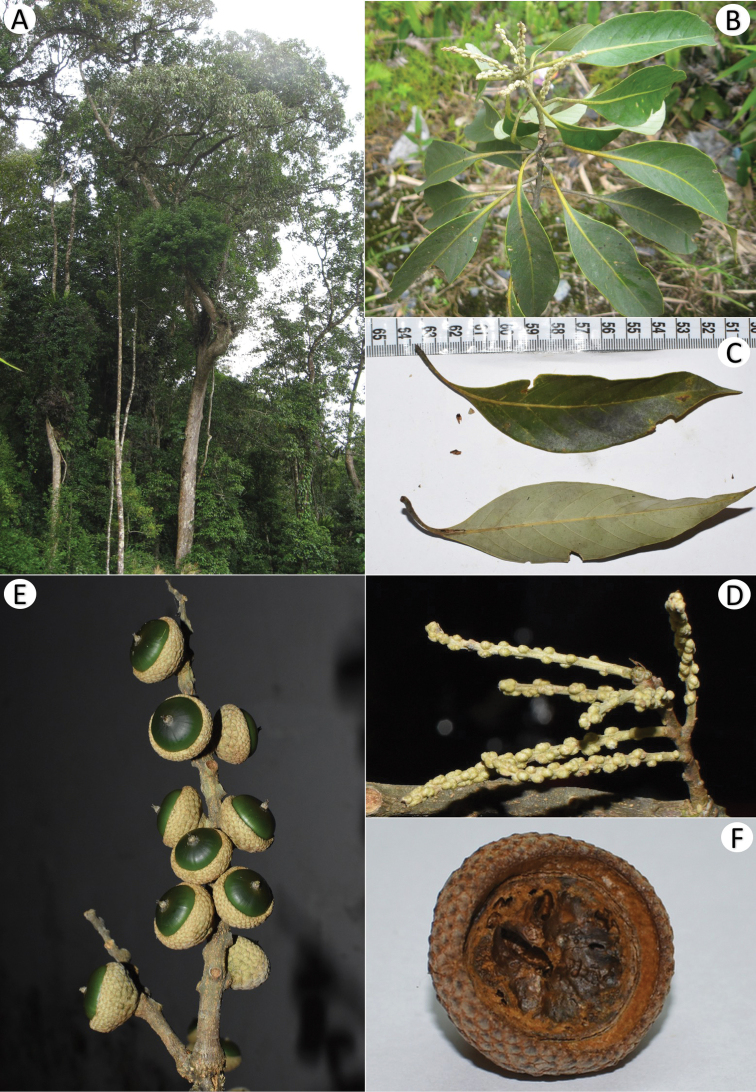
*Lithocarpushongiaoensis* Ngoc & Binh **A** habit **B** twig with young inflorescences **C** leaves **D** young male inflorescence **E** infructescence **F** inside of mature cupule.

##### Description.

Evergreen tree, up to 25 m tall. Twigs blackish gray, glabrescent, densely lenticellate. Stipules narrowly triangular, ca. 5 × 1 mm, densely covered with indumentum abaxially, almost glabrous adaxially. Leaves alternate; petioles 1.7–3.5 cm long, glabrous; blades narrowly elliptic to lanceolate, 7.6–14.7 × 2.3–4.6 cm, coriaceous, glabrous adaxially, covered with adherent waxy scales abaxially, apex acuminate, acumen up to 1.5 cm long, base attenuate and decurrent on petiole, margin entire, midrib flat or slightly prominent near base adaxially, prominent abaxially, greenish yellow *in vivo*, reddish brown *in sicco*; secondary veins 8–11 pairs, prominent abaxially, at an angle of 35–45° from the midrib, tertiary veins scalariform, faintly visible or invisible on both sides. Young male inflorescences terminal, ca. 5–7 cm long, densely covered with white indumentum. Infructescences terminal, erect, 12.5–16.5 cm long, rachis 0.4–0.6 cm thick at base, grayish brown, lenticellate, covered with indumentum. Cupules solitary, sessile, obconical to saucer-shaped, 0.8–1.2 cm high, 1.7–2.6 cm in diam., enclosing 1/3–1/2 of the nut; wall woody, ca. 2 mm thick, with triangular scales not united into concentric rings; scales up to 4 mm long, apex shortly acuminate, covered with dense grayish indumentum outside. Nut strongly depressed ovoid, 0.6–1.1 cm high, 1.2–1.5 cm in diam., glabrous, reddish brown to grayish brown *in sicco*, tomentose with soft white hairs near apex; basal scar slightly concave, 1.2–1.4 cm in diam.

##### Phenology.

Young male flowers and mature fruits were collected in May and June.

##### Distribution.

Vietnam (so far known only from Hon Giao area of Bidoup-Nui Ba National Park, Lam Dong Province). (Fig. 1).

##### Etymology.

The specific epithet is derived from the type locality, Hon Giao area of Bidoup-Nui Ba National Park, Lam Dong Province, Vietnam.

##### Local name.

Dé đá Hòn Giao.

##### Conservation status.

The new species is narrowly endemic to Hon Giao area of Bidoup-Nui Ba National Park, Lam Dong Province. During our floristic expedition from 2015 to present, we found only five mature individuals of *Lithocarpushongiaoensis* at the road in montane evergreen forest which is located within the protected area of the national park. According to criterion D of the IUCN Red List criteria (IUCN 2012), this species is qualified as CR.

##### Additional specimens examined.

Vietnam. Lam Dong Province: Bidoup-Nui Ba National Park, Hon Giao, 1574 m elev., 12°10'34.5"N, 108°42'25.5"E, 15 May 2019, *N.V. Ngoc*, *H.T. Binh*, *N.V. Duy*, *T.T. Nhung NAF122* [fr.] (DLU!); ibid., 1574 m elev., 12°10'35.5"N, 108°42'25.9"E, 11 June 2020, *N.V. Ngoc*, *H.T. Binh*, *N.V. Duy*, *T.T. Nhung NAF192* [fr.] (DLU!).

## Supplementary Material

XML Treatment for Lithocarpus bidoupensis

XML Treatment for Lithocarpus congtroiensis

XML Treatment for Lithocarpus hongiaoensis
